# Associations Between a Surrogate Index of Insulin Resistance and Hyperuricemia in Young and Middle‐Aged Patients With Type 2 Diabetes Mellitus

**DOI:** 10.1155/jdr/6682372

**Published:** 2026-07-02

**Authors:** Hongping Wang, Yunyi Yang, Jie Gao, Tao Lei, Juan Xia, Cuiping Zhang, Tian Shen, Jun Lu, Zongjun Liu

**Affiliations:** ^1^ Department of Endocrinology, Putuo Hospital, Shanghai University of Traditional Chinese Medicine, Shanghai, China, shutcm.edu.cn; ^2^ Yueyang Hospital of Integrated Traditional Chinese and Western Medicine, Shanghai University of Traditional Chinese Medicine, Shanghai, China, shutcm.edu.cn; ^3^ Department of Cardiology, Putuo Hospital, Shanghai University of Traditional Chinese Medicine, Shanghai, China, shutcm.edu.cn

**Keywords:** hyperuricemia, insulin resistance, Type 2 diabetes mellitus

## Abstract

**Background:**

This study is aimed at examining the associations between insulin resistance (IR) surrogate indices, including TyG, TyG‐BMI, TG/HDL‐C, and METS‐IR, and hyperuricemia (HUA) in young and middle‐aged patients with Type 2 diabetes mellitus (T2DM).

**Methods:**

A total of 1005 patients with T2DM were included in this cross‐sectional study. Participants were classified into HUA and nonhyperuricemia (NHUA) groups according to serum uric acid concentrations and further stratified by age into young (18–44 years) and middle‐aged (45–59 years) subgroups. Multivariable logistic regression analyses were performed to assess the associations between IR surrogate indices and HUA. Receiver operating characteristic (ROC) curve analysis was conducted to evaluate the discriminatory ability of these indices for HUA across sex‐ and age‐specific subgroups.

**Results:**

All IR surrogate indices were significantly higher in the HUA group than in the NHUA group. TyG and TG/HDL‐C showed stable associations with HUA across the regression models, while the association for TyG‐BMI was attenuated after additional adjustment when modeled continuously, although individuals in the highest quartile remained associated with higher odds of HUA. METS‐IR was associated with HUA only in unadjusted models. Age‐stratified analyses showed that none of the IR surrogate indices remained significantly associated with HUA after multivariable adjustment in younger patients, whereas TyG and TG/HDL‐C remained associated with higher odds of HUA in middle‐aged patients. ROC analyses indicated limited to moderate discriminatory performance (AUC < 0.75). TyG‐BMI showed the highest AUC in younger patients (AUC = 0.715), while TG/HDL‐C demonstrated relatively higher specificity in middle‐aged patients. Sex‐stratified analyses showed slightly higher AUC values in males than in females, although overall discrimination remained modest.

**Conclusion:**

IR surrogate indices showed varying associations with HUA in young and middle‐aged patients with Type 2 diabetes. TyG and TG/HDL‐C showed relatively stable associations with HUA across the regression models, whereas the associations for TyG‐BMI and METS‐IR were attenuated after additional adjustment. Overall, these indices demonstrated limited discriminatory performance and are not suitable as standalone diagnostic tools but may serve as adjunctive markers for preliminary risk stratification.

## 1. Introduction

Hyperuricemia (HUA) is a chronic metabolic disorder characterized by abnormally elevated serum uric acid (SUA) levels, resulting from excessive uric acid production and/or impaired renal excretion [1]. HUA is closely linked to gout and chronic kidney disease [2–4] and is also associated with a broad spectrum of metabolic disorders, including cardiovascular disease (CVD), obesity, Type 2 diabetes mellitus (T2DM), metabolic syndrome, and hypertension [5–9]. Experimental and clinical studies have suggested that elevated SUA levels are involved in multiple pathophysiological processes, such as the induction of inflammatory responses, endothelial dysfunction, vascular smooth muscle cell proliferation, and activation of the renin–angiotensin system. In addition, increased expression of uric acid transporters, including urate Transporter 1 (URAT1) and glucose Transporter 9 (GLUT9), as well as alterations in glycolytic pathways related to insulin resistance (IR), has been implicated in uric acid homeostasis [10]. The global prevalence of HUA has increased substantially in recent decades, posing a major public health challenge. The prevalence exceeds 20% in the United States [11], is approximately 16.6% in South Australia [12], and reaches 13.3% in China [13], ranking fourth among metabolic disorders after diabetes, hypertension, and hyperlipidemia. With population aging and ongoing lifestyle changes, the burden of HUA is expected to continue rising. Elevated SUA levels have been associated with a higher prevalence of complications and increased all‐cause mortality among patients with diabetes [14], and HUA‐related vascular damage may be difficult to reverse [15]. These observations highlight the clinical and public health importance of identifying factors associated with HUA, particularly in high‐risk populations.

IR, defined as reduced responsiveness of target tissues to insulin‐mediated metabolic actions, represents a core pathophysiological feature of T2DM [16] and is also involved in the development of multiple metabolic disorders [17]. Previous studies have demonstrated that states of IR or hyperinsulinemia are associated with increased renal tubular reabsorption of sodium and uric acid, resulting in higher SUA levels [18]. Moreover, reduced renal uric acid clearance under hyperinsulinemic conditions has been linked to disturbances in purine metabolism and altered uric acid handling [19]. Collectively, these findings support a close association between IR and elevated SUA levels. Mendelian randomization studies have further suggested a relationship between improvements in insulin sensitivity and lower SUA concentrations, as well as reduced gout susceptibility [20]. Taken together, existing evidence indicates that IR‐related metabolic alterations are closely linked to uric acid metabolism.

Although the hyperinsulinemic–euglycemic clamp remains the gold standard for assessing IR [21], its complexity, cost, and invasiveness limit its feasibility in routine clinical practice [22]. While the homeostasis model assessment of insulin resistance (HOMA‐IR) is commonly used, its reliance on insulin measurements and issues related to standardization has encouraged the development of simpler surrogate indices. Several noninsulin‐based IR indices derived from routinely available clinical parameters have therefore been proposed, including the triglyceride–glucose (TyG) index [23], TyG combined with body mass index (TyG‐BMI) [24], the triglyceride‐to‐high‐density lipoprotein cholesterol ratio (TG/HDL‐C) [25], and the metabolic score for insulin resistance (METS‐IR) [26]. These indices have shown acceptable performance for reflecting IR in clinical and epidemiological settings and are increasingly used as pragmatic markers of metabolic dysfunction.

However, data remain limited regarding the associations between IR surrogate indices and HUA among young and middle‐aged patients with T2DM. Therefore, the present study is aimed at examining the relationships between several commonly used IR surrogate indices (TyG, TyG‐BMI, TG/HDL‐C, and METS‐IR) and HUA in this population. By evaluating these associations across age and sex subgroups, this study seeks to provide further evidence to inform metabolic risk assessment and clinical management strategies for HUA in patients with T2DM.

## 2. Materials and Methods

### 2.1. Study Design and Population

**Figure 1:**
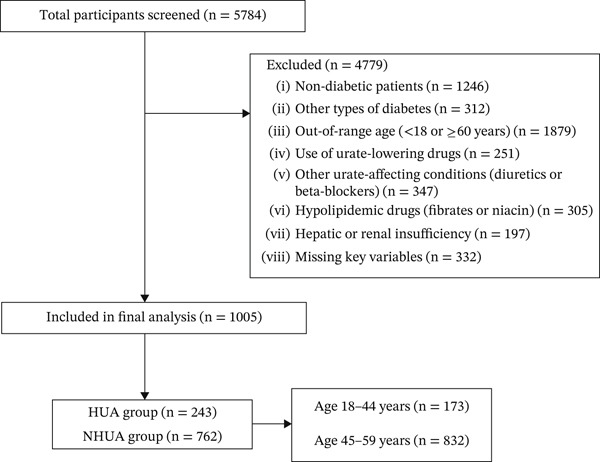
Flowchart of participant selection for the study.

In this cross‐sectional study, we included 1005 patients with T2DM who were admitted to the endocrine ward of Putuo Hospital, Shanghai University of Traditional Chinese Medicine, between January 2016 and December 2019. A total of 5784 participants were initially screened. After excluding individuals with missing key variables, non–Type 2 diabetes, out‐of‐range age (< 18 or ≥ 60 years), hepatic or renal insufficiency, use of urate‐lowering or urate‐altering medications, and other conditions known to affect uric acid metabolism, 1005 patients with T2DM were included in the final analysis. The participant selection process is illustrated in Figure 1.

The inclusion criteria were as follows: (1) fulfillment of the diagnostic criteria outlined in the Chinese Guidelines for the Prevention and Control of Type 2 Diabetes Mellitus (2020 edition) [27] and (2) an age range of 18–59 years.

The exclusion criteria were as follows: (1) diagnosis of other types of diabetes mellitus, such as Type 1 diabetes, (2) use of medications within the past 3 months that could potentially influence uric acid production and metabolism, (3) presence of hepatic or renal insufficiency, and (4) other conditions that could affect uric acid levels, including nephritis, renal failure, severe liver disease, thyroid dysfunction, tumors, or infectious diseases.

Specifically, patients receiving urate‐lowering or urate‐altering drugs (such as febuxostat, allopurinol, benzbromarone, or uricase‐based agents), diuretics (including hydrochlorothiazide, furosemide, bumetanide, or indapamide), or *β*‐blockers (such as propranolol or metoprolol) were excluded. Among lipid‐lowering therapies, individuals currently using fibrates or niacin were also excluded. However, some patients receiving low‐dose aspirin, statins, or sodium–glucose cotransporter‐2 (SGLT2) inhibitors were not excluded due to their common clinical use in diabetes management. To account for potential confounding, these medications were incorporated as covariates in the adjusted regression model.

Based on the diagnostic criteria for HUA outlined in the Chinese Guidelines for the Diagnosis and Treatment of Hyperuricemia and Gout (2019 edition) [28], HUA was defined as fasting SUA > 420 *μ*mol/L. SUA measurements were obtained from routine clinical laboratory testing recorded in the hospital electronic medical record system, and the baseline fasting SUA value at admission was used for the primary analyses. Accordingly, participants were classified into the HUA group (SUA > 420 *μ*mol/L, *n* = 243) and the nonhyperuricemia (NHUA) group (SUA ≤ 420 *μ*mol/L, *n* = 762). To account for potential sex‐specific physiological differences in SUA levels, a sensitivity analysis was additionally performed using sex‐specific cutoff values, defined as > 360 *μ*mol/L for women and > 420 *μ*mol/L for men, as commonly adopted in epidemiological studies. Key analyses were repeated under this alternative definition to assess the robustness of the primary findings. According to the World Health Organization (WHO) criteria [29] and age stratification commonly adopted in previous studies on diabetes and metabolic disorders [30, 31], participants were further categorized into young (18–44 years) and middle‐aged (45–59 years) groups based on age.

The study protocol complied with the ethical principles of the Declaration of Helsinki and was approved by the Research Ethics Committee of Putuo Hospital, Shanghai University of Traditional Chinese Medicine (Ethics Code: PTEC‐A‐2024‐73(S)‐1). This study was a retrospective analysis of clinical data collected between 2016 and 2019, and ethical approval was obtained retrospectively prior to data analysis. Written informed consent had been obtained from all participants at the time of data collection.

### 2.2. Data Collection and Measurement

General patient information collected included age, gender, and medical history. The specific definitions for each parameter were as follows:

• Duration of T2DM: Defined as the cumulative number of years from the diagnosis of T2DM to the date of the survey.
• History of hypertension (HBP): Participants with a documented HBP were classified as having a history of HBP; those without a documented history were considered not having hypertension.
• CVD: Participants self‐reported a diagnosis of heart disease by a healthcare provider.

For physical measurements, patients were instructed to stand barefoot on a multifunctional weight scale, which automatically recorded their weight and calculated BMI as weight (kilogram) divided by the square of height (square meter). Blood pressure (BP) was measured by a trained medical professional between 8:00 and 9:00 a.m., after the patient had rested for 20 min. Three consecutive BP readings were taken and averaged, with results recorded as systolic blood pressure (SBP) and diastolic blood pressure (DBP) in millimeters of mercury.

Fasting blood samples were collected by medical professionals for biochemical analysis. Key indices, including fasting blood glucose (FBG), 2‐h postprandial blood glucose (2h‐BG), total cholesterol (TC), low‐density lipoprotein cholesterol (LDL‐C), high‐density lipoprotein cholesterol (HDL‐C), and triglycerides (TGs), were measured using the enzyme colorimetric method. Glycated hemoglobin (HbA1c) was assessed by high‐performance liquid chromatography. Fasting insulin (F‐ins) and fasting C‐peptide (F‐CP) levels were measured by chemiluminescence. HOMA‐IR was calculated using the following formula: HOMA − IR = FPG (mmol/L) × FINS (mU/L)/22.5.

Renal function was assessed with serum creatinine (SCr) measured by the rate blank method and the compensated Jaffe′s method. Blood urea nitrogen (BUN) was determined using the urease‐activated ultraviolet spectrophotometric method (UV‐enzyme reaction). SUA was measured by the UA‐UV method. The estimated glomerular filtration rate (eGFR) was calculated using the CKD‐EPI formula [32].

This data collection and measurement process followed standardized operating procedures to ensure the accuracy and consistency of the results.

### 2.3. IR Index Calculation

The formulas for the four IR proxies (TyG, TyG‐BMI, TG/HDL‐C, and METS‐IR) are provided as follows:

1. TyG = ln[TG (mg/dL) × FPG (mg/dL)/2] [33].
2. TyG − BMI = TyG × BMI (kg/m^2^).
3. TG/HDL − C = TG (mg/dL)/HDL − C (mg/dL) [34].
4. METS − IR = ln[2 × FPG (mg/dL) + TG (mg/dL)] × BMI (kg/m^2^)/ln[HDL − C (mg/dL)] [35].

To ensure transparency and reproducibility, a worked example of the calculation process is provided in Supporting Information 1: Table S1.

### 2.4. Statistical Analysis

Data were analyzed using IBM SPSS 27.0 software. Continuous variables that followed a normal distribution were expressed as the mean ± standard deviation ($\bar{x}\pm s$), and comparisons between groups were conducted using an independent samples *t*‐test. Continuous variables with a nonnormal distribution are reported as the median (25th–75th percentile), and group comparisons were made using the Mann–Whitney *U* test. Qualitative data were expressed as frequencies (percentages), and comparisons between groups were performed using the *χ* ^2^ test.

Logistic regression analyses were performed to examine the associations between IR‐related surrogate indices and HUA. Odds ratios (ORs) with 95% confidence intervals (CIs) were estimated using unadjusted and multivariable‐adjusted models. Renal function and liver enzymes are closely related to both IR and uric acid metabolism and may reflect underlying metabolic status. Therefore, a minimally adjusted model including age, sex, diabetes duration, HBP, and history of CVD was specified as the primary analytical model. An exploratory model additionally including hepatic and renal markers (ALT, AST, and eGFR) was fitted to assess the robustness of the observed associations under more extensive metabolic adjustment. Receiver operating characteristic (ROC) curve analysis was conducted to evaluate the discriminatory ability of each IR surrogate index for HUA. The area under the ROC curve (AUC) was calculated, and sensitivity, specificity, and optimal cutoff values were determined using the Youden index (sensitivity + specificity − 1). For internal validation, logistic regression models were constructed using each IR surrogate index as the predictor, and model calibration was assessed using the Hosmer–Lemeshow goodness‐of‐fit test. Bootstrap resampling with 1000 iterations was performed to evaluate the stability of model performance, and bias‐corrected and accelerated (BCa) 95% CIs for the AUC were calculated based on predicted probabilities derived from the fitted logistic regression models.

Missing data were handled using listwise deletion, as the proportion of missing values was less than 5% for all key variables; therefore, no imputation was performed. Subgroup analyses stratified by age and sex were performed to further examine associations between IR surrogate indices and HUA, as well as differences in discriminatory ability across subgroups. A two‐sided *p* value < 0.05 was considered statistically significant.

## 3. Results

### 3.1. Baseline Characteristics

The baseline characteristics of 1005 young and middle‐aged patients with T2DM, categorized by SUA levels, are summarized in Table 1. Patients in the HUA group were significantly younger (*p* < 0.001) and had a shorter duration of diabetes compared to those in the NHUA group (*p* = 0.008). The proportion of males was higher in the HUA group (82.72% vs. 62.47%, *p* < 0.001). No significant differences were observed between the two groups in the prevalence of HBP or CVD (*p* = 0.208 and *p* = 0.580, respectively). Regarding medication use, there were no significant differences between the two groups in the use of low‐dose aspirin or statins (both *p* = 0.931). However, the proportion of patients using SGLT2 inhibitors was significantly higher in the HUA group compared with the NHUA group (13.99% vs. 7.74%, *p* = 0.003). Biochemical indices revealed that F‐CP, F‐ins, BUN, SCr, TC, and TG were significantly elevated in the HUA group compared to the NHUA group (*p* < 0.001). In contrast, the eGFR was significantly lower in the HUA group (*p* < 0.001), and HDL‐C levels were significantly lower (*p* = 0.034). IR proxies, including HOMA‐IR, TyG, TyG‐BMI, TG/HDL‐C, and METS‐IR, were all significantly higher in the HUA group (*p* < 0.001), indicating a strong correlation between IR and HUA.

**Table 1:** Baseline characteristics of subjects stratified by hyperuricemia status.

Variables	Total (*n* = 1005)	NHUA (*n* = 762)	HUA (*n* = 243)	*p*
Age (years)	55.00 (49.00, 58.00)	56.00 (50.00, 58.75)	53.00 (43.00, 58.00)	< 0.001
Diabetes duration (years)	6.00 (1.00, 10.00)	7.00 (2.00, 10.00)	5.00 (0.45, 10.00)	0.008
Age, *n* (%)				< 0.001
18–44 years	173 (17.22)	108 (62.43)	65 (37.57)	
45–59 years	832 (82.78)	655 (78.47)	178 (21.53)	
Sex, *n* (%)				< 0.001
Male	677 (67.36)	476 (62.47)	201 (82.72)	
Female	328 (32.64)	286 (37.53)	42 (17.28)	
HBP, *n* (%)				0.208
Yes	457 (45.47)	338 (44.36)	119 (48.97)	
No	548 (54.53)	424 (55.64)	124 (51.03)	
CVD, *n* (%)				0.580
Yes	156 (15.52)	121 (15.88)	35 (14.40)	
No	849 (84.48)	641 (84.12)	208 (85.60)	
Low‐dose aspirin, *n* (%)				0.931
Yes	134 (13.33)	102 (13.39)	32 (13.17)	
No	871 (86.67)	660 (86.61)	211 (86.83)	
Statins, *n* (%)				0.931
Yes	134 (13.33)	102 (13.39)	32 (13.17)	
No	871 (86.67)	660 (86.61)	211 (86.83)	
SGLT2 inhibitors				0.003
Yes	93 (9.25)	59 (7.74)	34 (13.99)	
No	912 (90.75)	703 (92.26)	209 (86.01)	
HbA1c	9.50 (8.00, 11.20)	9.40 (8.00, 11.00)	9.80 (7.73, 11.70)	0.305
FBG (mmol/L)	8.85 (6.60, 11.80)	8.70 (6.60, 11.50)	9.50 (6.65, 12.40)	0.089
2h‐BG (mmol/L)	15.40 (11.60, 19.00)	15.10 (11.43, 18.80)	16.10 (12.00, 19.35)	0.142
F‐CP (ng/mL)	1.17 (0.71, 1.82)	1.11 (0.68, 1.71)	1.43 (0.81, 2.26)	< 0.001
F‐ins (mU/L)	9.45 (5.44, 14.53)	8.84 (5.19, 13.87)	11.66 (6.88, 16.82)	< 0.001
BUN (mmol/L)	5.70 (4.70, 6.90)	5.60 (4.70, 6.60)	6.70 (4.90, 8.70)	< 0.001
SCr (*μ*mol/L)	62.00 (51.00, 74.00)	60.00 (50.00, 70.00)	76.00 (60.00, 98.00)	< 0.001
eGFR (mL/min/1.73 m^2^)	111.52 (93.13, 135.77)	114.55 (96.93, 136.94)	99.82 (63.80, 128.36)	< 0.001
ALT (U/L)	20.00 (14.00, 32.00)	20.00 (14.00, 31.00)	24.00 (15.00, 38.00)	0.001
AST (U/L)	19.00 (16.00, 26.00)	19.00 (15.75, 25.00)	22.00 (17.00, 31.00)	< 0.001
TC (mmol/L)	4.75 (3.93, 5.63)	4.69 (3.89, 5.55)	5.00 (4.09, 6.01)	0.003
TG (mmol/L)	1.74 (1.15, 2.64)	1.65 (1.09, 2.41)	2.27 (1.49, 3.94)	< 0.001
HDL‐C (mmol/L)	0.99 (0.84, 1.19)	1.00 (0.86, 1.19)	0.95 (0.80, 1.20)	0.034
LDL‐C (mmol/L)	3.08 (2.50, 3.71)	3.08 (2.49, 3.64)	3.08 (2.61, 3.88)	0.118
BMI (kg·[m^2^]^−1^)	24.61 (22.20, 27.04)	24.39 (22.03, 26.67)	25.39 (23.02, 28.71)	< 0.001
HOMA‐IR	3.47 (2.11, 6.10)	3.27 (2.05, 5.58)	4.64 (2.55, 8.44)	< 0.001
TyG	7.84 (7.23, 8.41)	7.75 (7.21, 8.31)	8.20 (7.61, 8.89)	< 0.001
TyG‐BMI	190.58 (165.41, 222.83)	186.82 (163.34, 213.25)	207.39 (185.23, 250.24)	< 0.001
TG/HDL‐C	1.78 (1.07, 2.84)	1.66 (1.00, 2.51)	2.54 (1.40, 4.57)	< 0.001
METS‐IR	49.18 (43.25, 57.27)	48.56 (42.96, 55.51)	52.59 (45.76, 63.84)	< 0.001

*Note:* Unit conversion: FPG (mg/dL)/18 = mmol/L; TG (mg/dL)/88.57 = mmol/L; HDL‐C (mg/dL)/38.67 = mmol/L. Data are presented as median (IQR) for continuous variables and number (proportion) for categorical variables. Differences in medians were examined using the Mann–Whitney test between two groups; differences in proportions were tested using the chi‐square test.

Abbreviations: 2h‐BG, 2‐h postprandial blood glucose; BMI, body mass index; BUN, blood urea nitrogen; CVD, cardiovascular disease; eGFR, estimated glomerular filtration rate; FBG, fasting blood glucose; F‐CP, fasting C‐peptide; F‐ins, fasting insulin; HbA1c, glycated hemoglobin; HBP, history of hypertension; HDL‐C, high‐density lipoprotein cholesterol; HOMA‐IR, homeostasis model assessment of insulin resistance; LDL‐C, low‐density lipoprotein cholesterol; TC, total cholesterol; TG, triglyceride.

Table 2 presents the baseline characteristics of T2DM patients aged 18–44 years. Patients in the HUA group were significantly younger than those in the NHUA group (*p* = 0.002) and had a shorter duration of diabetes (*p* = 0.006). Additionally, the proportion of males was significantly higher in the HUA group (*p* < 0.001). Regarding the HBP, the proportion of patients in the HUA group was slightly higher than in the NHUA group (*p* = 0.017), while the distribution of CVD between the two groups showed no significant difference (*p* = 0.991). With respect to medication use, there were no significant differences between the two groups in the use of low‐dose aspirin (*p* = 0.140) or statins (*p* = 0.140). The proportion of patients treated with SGLT2 inhibitors was higher in the HUA group compared with the NHUA group (18.46% vs. 10.19%), although this difference did not reach statistical significance (*p* = 0.120). Patients in the HUA group had higher levels of FBG, 2h‐BG, F‐CP, and F‐ins compared to the NHUA group, with significant differences observed for F‐CP and F‐ins (*p* < 0.05). SCr, ALT, and AST levels were also higher in the HUA group, while no significant differences were found in eGFR between the two groups. TG levels were significantly higher in the HUA group, while HDL‐C levels were lower, indicating the presence of lipid metabolism abnormalities in the HUA group. Furthermore, IR proxies, including HOMA‐IR, TyG, TyG‐BMI, METS‐IR, and TG/HDL‐C, were significantly elevated in the HUA group, suggesting a close association between IR and HUA in T2DM patients aged 18–44 years.

**Table 2:** Baseline characteristics of patients aged 18–44 years.

Variables	Total (*n* = 173)	NHUA (*n* = 108)	HUA (*n* = 65)	*p*
Age (years)	37.00 (32.00, 40.00)	37.50 (33.00, 41.00)	35.00 (29.00, 39.00)	0.002
Diabetes duration (years)	1.00 (0.10, 4.00)	2.00 (0.18, 5.25)	0.50 (0.10, 2.00)	0.006
Sex, *n* (%)				< 0.001
Male	140 (80.92)	83 (76.85)	57 (87.69)	
Female	33 (19.08)	25 (23.15)	8 (12.31)	
HBP, *n* (%)				0.017
Yes	37 (21.39)	22 (20.37)	15 (23.08)	
No	136 (78.61)	86 (79.63)	50 (76.92)	
CVD, *n* (%)				0.991
Yes	2 (1.16)	0 (0.00)	2 (3.08)	
No	171 (98.84)	108 (100.00)	63 (96.92)	
Low‐dose aspirin, *n* (%)				0.140
Yes	2 (1.16)	0 (0.00)	2 (3.08)	
No	171 (98.84)	108 (100.00)	63 (96.92)	
Statins, *n* (%)				0.140
Yes	2 (1.16)	0 (0.00)	2 (3.08)	
No	171 (98.84)	108 (100.00)	63 (96.92)	
SGLT2 inhibitors				0.120
Yes	23 (13.29)	11 (10.19)	12 (18.46)	
No	150 (86.71)	97 (89.81)	53 (81.54)	
HbA1c	11.00 (9.10, 12.30)	10.80 (8.90, 12.00)	11.40 (10.10, 12.75)	0.065
FBG (mmol/L)	10.45 (7.90, 13.53)	10.00 (7.48, 13.25)	11.05 (8.40, 13.78)	0.171
2h‐BG (mmol/L)	16.20 (11.95, 19.08)	16.25 (10.83, 18.83)	16.15 (12.93, 19.75)	0.347
F‐CP (ng/mL)	1.15 (0.78, 1.85)	1.03 (0.76, 1.65)	1.53 (0.84, 2.13)	0.026
F‐ins (mU/L)	9.80 (5.08, 14.41)	8.20 (4.38, 12.27)	12.77 (7.75, 17.83)	0.001
BUN (mmol/L)	4.90 (4.10, 6.00)	4.90 (4.05, 5.80)	5.00 (4.30, 6.27)	0.236
SCr (*μ*mol/L)	58.50 (50.00, 69.75)	58.00 (49.00, 66.00)	63.00 (53.25, 79.50)	0.018
eGFR (mL/min/1.73 m^2^)	137.13 (114.61, 163.34)	137.40 (121.57, 163.30)	134.24 (110.03, 163.77)	0.376
ALT (U/L)	28.50 (16.00, 47.00)	26.50 (13.25, 42.00)	37.00 (21.50, 61.25)	0.025
AST (U/L)	23.00 (16.00, 31.25)	20.00 (15.00, 28.00)	25.00 (19.00, 40.50)	0.011
TC (mmol/L)	5.04 (4.17, 5.86)	5.04 (4.15, 5.87)	5.05 (4.21, 5.83)	0.707
TG (mmol/L)	2.29 (1.58, 3.66)	2.15 (1.46, 2.94)	2.75 (1.98, 4.48)	0.004
HDL‐C (mmol/L)	0.86 (0.78, 1.01)	0.89 (0.81, 1.02)	0.82 (0.72, 0.98)	0.041
LDL‐C (mmol/L)	3.34 (2.71, 3.96)	3.38 (2.72, 4.00)	3.25 (2.70, 3.87)	0.751
BMI (kg·[m^2^]^−1^)	25.81 (23.67, 30.52)	24.49 (22.67, 27.57)	28.72 (24.85, 33.03)	< 0.001
HOMA‐IR	4.02 (2.38, 6.63)	3.16 (2.26, 5.89)	6.00 (3.55, 9.05)	< 0.001
TyG	8.44 (7.78, 8.81)	8.18 (7.51, 8.62)	8.63 (8.09, 9.13)	0.004
TyG‐BMI	217.41 (184.21, 271.96)	207.65 (176.11, 245.35)	255.32 (219.73, 307.48)	< 0.001
TG/HDL‐C	2.75 (1.73, 4.31)	2.21 (1.61, 3.62)	3.73 (2.41, 5.60)	< 0.001
METS‐IR	58.31 (48.96, 71.32)	53.54 (47.03, 62.46)	67.71 (56.85, 76.33)	< 0.001

*Note:* Data are presented as median (IQR) for continuous variables and number (proportion) for categorical variables. Differences in medians were examined using the Mann–Whitney test between two groups; differences in proportions were tested using the chi‐square test.

Abbreviations: 2h‐BG, 2‐h postprandial blood glucose; BMI, body mass index; BUN, blood urea nitrogen; CVD, cardiovascular disease; eGFR, estimated glomerular filtration rate; FBG, fasting blood glucose; F‐CP, fasting C‐peptide; F‐ins, fasting insulin; HbA1c, glycated hemoglobin; HBP, history of hypertension; HDL‐C, high‐density lipoprotein cholesterol; HOMA‐IR, homeostasis model assessment of insulin resistance; LDL‐C, low‐density lipoprotein cholesterol; TC, total cholesterol; TG, triglyceride.

Table 3 presents the baseline characteristics of patients aged 45–59 years. The mean age of patients in the HUA group was slightly lower than that in the NHUA group (*p* = 0.014), and the proportion of males was significantly higher in the HUA group (*p* < 0.001). Additionally, hypertension was significantly more prevalent in the HUA group compared to the NHUA group (*p* = 0.017). With respect to medication use, there were no significant differences between the two groups in the use of low‐dose aspirin or statins (both *p* = 0.684). However, a significantly higher proportion of patients in the HUA group were treated with SGLT2 inhibitors compared to the NHUA group (12.36% vs. 7.34%, *p* = 0.032). Among the biochemical indices, significant differences were observed in F‐ins, BUN, SCr, eGFR, AST, TC, and TG in middle‐aged HUA patients (*p* < 0.05). Furthermore, IR proxies, including HOMA‐IR, TyG, TyG‐BMI, TG/HDL‐C, and METS‐IR, were significantly higher in the HUA group than in the NHUA group (*p* < 0.001), consistent with a higher prevalence of IR in patients with HUA.

**Table 3:** Baseline characteristics of patients aged 45‐59 years.

Variables	Total (*n* = 832)	NHUA (*n* = 654)	HUA (*n* = 178)	*p*
Age (years)	57.00 (53.00, 59.00)	57.00 (54.00, 59.00)	56.00 (51.00, 58.00)	0.014
Diabetes duration (years)	8.00 (2.00, 12.00)	8.00 (2.00, 12.00)	8.00 (2.00, 13.75)	0.968
Sex, *n* (%)				< 0.001
Male	537 (64.54)	393 (60.09)	144 (80.90)	
Female	295 (35.46)	261 (39.91)	34 (19.10)	
HBP, *n* (%)				0.017
Yes	420 (50.48)	316 (48.32)	104 (58.43)	
No	412 (49.52)	338 (51.68)	74 (41.57)	
CVD, *n* (%)				0.991
Yes	154 (18.51)	121 (18.50)	33 (18.54)	
No	678 (81.49)	533 (81.50)	145 (81.46)	
Low‐dose aspirin, *n* (%)				0.684
Yes	132 (15.87)	102 (15.60)	30 (16.85)	
No	700 (84.13)	552 (84.40)	148 (83.15)	
Statins, *n* (%)				0.684
Yes	132 (15.87)	102 (15.60)	30 (16.85)	
No	700 (84.13)	552 (84.40)	148 (83.15)	
SGLT2 inhibitors				0.032
Yes	70 (8.41)	48 (7.34)	22 (12.36)	
No	762 (91.59)	606 (92.66)	156 (87.64)	
HbA1c	9.30 (7.90, 10.90)	9.40 (7.95, 10.90)	9.05 (7.40, 10.90)	0.339
FBG (mmol/L)	8.60 (6.50, 11.50)	8.60 (6.60, 11.50)	8.70 (6.40, 11.80)	0.637
2h‐BG (mmol/L)	15.10 (11.60, 18.95)	15.00 (11.50, 18.80)	16.00 (12.00, 19.30)	0.312
F‐CP (ng/mL)	1.17 (0.70, 1.80)	1.13 (0.68, 1.71)	1.40 (0.81, 2.30)	0.003
F‐ins (mU/L)	9.40 (5.48, 14.63)	8.92 (5.28, 14.27)	11.36 (6.84, 16.42)	0.002
BUN (mmol/L)	5.80 (4.90, 7.10)	5.70 (4.80, 6.70)	7.50 (5.60, 9.70)	< 0.001
SCr (*μ*mol/L)	62.00 (52.00, 75.00)	60.00 (51.00, 70.00)	79.00 (67.50, 111.50)	< 0.001
eGFR (mL/min/1.73 m^2^)	106.80 (89.67, 128.50)	110.36 (95.13, 130.98)	91.01 (57.10, 106.39)	< 0.001
ALT (U/L)	20.00 (14.00, 30.00)	19.00 (14.00, 29.00)	21.00 (15.00, 34.00)	0.113
AST (U/L)	19.00 (16.00, 25.00)	19.00 (16.00, 24.00)	21.00 (16.00, 28.00)	0.022
TC (mmol/L)	4.68 (3.89, 5.56)	4.64 (3.84, 5.45)	4.94 (4.08, 6.04)	0.006
TG (mmol/L)	1.65 (1.09, 2.46)	1.58 (1.07, 2.25)	1.98 (1.40, 3.75)	< 0.001
HDL‐C (mmol/L)	1.01 (0.87, 1.22)	1.01 (0.87, 1.21)	1.01 (0.86, 1.24)	0.696
LDL‐C (mmol/L)	3.05 (2.48, 3.66)	3.04 (2.46, 3.61)	3.05 (2.59, 3.89)	0.174
BMI (kg·[m^2^]^−1^)	24.49 (22.05, 26.69)	24.24 (21.97, 26.67)	24.91 (22.41, 27.41)	0.061
HOMA‐IR	3.42 (2.09, 5.86)	3.32 (2.02, 5.50)	4.24 (2.41, 7.29)	0.011
TyG	7.75 (7.20, 8.30)	7.70 (7.18, 8.23)	8.07 (7.39, 8.60)	< 0.001
TyG‐BMI	187.49 (163.34, 213.35)	185.58 (162.15, 210.93)	197.31 (181.39, 231.31)	< 0.001
TG/HDL‐C	1.64 (0.99, 2.57)	1.54 (0.96, 2.39)	2.09 (1.21, 3.85)	< 0.001
METS‐IR	48.29 (42.88, 54.95)	47.86 (42.66, 54.53)	49.62 (44.51, 58.21)	0.035

*Note:* Data are presented as median (IQR) for continuous variables and number (proportion) for categorical variables. Differences in medians were examined using the Mann–Whitney test between two groups; differences in proportions were tested using the chi‐square test.

Abbreviations: 2h‐BG, 2‐h postprandial blood glucose; BMI, body mass index; BUN, blood urea nitrogen; CVD, cardiovascular disease; eGFR, estimated glomerular filtration rate; FBG, fasting blood glucose; F‐CP, fasting C‐peptide; F‐ins, fasting insulin; HbA1c, glycated hemoglobin; HBP, history of hypertension; HDL‐C, high‐density lipoprotein cholesterol; HOMA‐IR, homeostasis model assessment of insulin resistance; LDL‐C, low‐density lipoprotein cholesterol; TC, total cholesterol; TG, triglyceride.

### 3.2. Associations Between IR Surrogates and HUA

Table 4 summarizes the associations between IR surrogate indices and HUA across three logistic regression models. ORs and 95% CIs were estimated for TyG, TyG‐BMI, TG/HDL‐C, and METS‐IR, both as continuous variables and by quartiles. Model 1 was unadjusted; Model 2 was adjusted for age, sex, disease duration, hypertension, and CVD; and Model 3 was further adjusted for hepatic and renal markers (ALT, AST, and eGFR). Higher TyG levels were associated with HUA across progressively adjusted models. In quartile analyses, participants in the highest TyG quartile (Q4) had higher odds of HUA compared with those in the lowest quartile (Q1) in Model 1 (OR = 3.292, 95% CI: 1.979–5.477, *p* < 0.001), Model 2 (OR = 2.490, 95% CI: 1.425–4.351, *p* < 0.001), and Model 3 (OR = 4.335, 95% CI: 1.833–10.249, *p* < 0.001). A significant dose–response trend across TyG quartiles was observed in all three models (all *p* for trend < 0.001). Supporting Information 2: Table S2 provides the variance inflation factors (VIFs) for all covariates included in Model 3 across the four IR surrogate indices, confirming minimal multicollinearity.

**Table 4:** Associations between quartiles of insulin resistance surrogate indices and hyperuricemia.

Variables	Model 1		Model 2		Model 3	
	OR (95% CI)	*p*	OR (95% CI)	*p*	OR (95% CI)	*p*
TyG	1.754 (1.437–2.141)	< 0.001	1.528 (1.237–1.907)	< 0.001	1.774 (1.270–2.478)	< 0.001
TyG Q1	1.00	—	1.00	—	1.00	—
TyG Q2	0.944 (0.524–1.703)	0.849	0.993 (0.541–1.823)	0.982	1.347 (0.564–3.219)	0.502
TyG Q3	1.776 (1.039–3.037)	0.036	1.720 (0.977–3.028)	0.060	2.276 (0.930–5.570)	0.071
TyG Q4	3.292 (1.979–5.477)	< 0.001	2.490 (1.425–4.351)	< 0.001	4.335 (1.833–10.249)	< 0.001
TyG group trend	2.059 (1.580–2.683)	< 0.001	1.723 (1.293–2.296)	< 0.001	2.274 (1.476–3.505)	< 0.001
TyG‐BMI	1.008 (1.004–1.012)	< 0.001	1.004 (1.000–1.007)	0.029	1.002 (0.998–1.006)	0.289
TyG‐BMI Q1	1.00	—	1.00	—	1.00	—
TyG‐BMI Q2	1.090 (0.613–1.940)	0.769	0.975 (0.539–1.764)	0.934	0.582 (0.237–1.427)	0.237
TyG‐BMI Q3	1.415 (0.814–2.460)	0.218	1.242 (0.705–2.188)	0.453	1.239 (0.539–2.845)	0.614
TyG‐BMI Q4	3.330 (2.000–5.546)	< 0.001	2.196 (1.244–3.877)	< 0.001	2.399 (1.011–5.697)	0.047
TyG‐BMI group trend	1.014 (1.009–1.019)	< 0.001	1.009 (1.003–1.015)	0.002	1.012 (1.003–1.021)	0.006
TG/HDL‐C	1.141 (1.081–1.205)	< 0.001	1.101 (1.043–1.163)	< 0.001	1.120 (1.023–1.225)	0.013
TG/HDL‐C Q1	1.00	—	1.00	—	1.00	—
TG/HDL‐C Q2	1.083 (0.622–1.887)	0.777	0.968 (0.547–1.713)	0.910	1.170 (0.490–2.795)	0.150
TG/HDL‐C Q3	1.625 (0.964–2.739)	0.068	2.430 (1.433–4.120)	0.455	3.219 (1.405–7.375)	0.005
TG/HDL‐C Q4	3.659 (2.248–5.956)	< 0.001	2.477 (1.479–4.149)	< 0.001	3.309 (1.427–7.671)	0.005
TG/HDL‐C group trend	1.456 (1.295–1.637)	< 0.001	1.315 (1.155–1.496)	< 0.001	1.378 (1.132–1.678	0.001
METS‐IR	1.026 (1.012–1.040)	< 0.001	1.008 (0.995–1.021)	0.239	1.001 (0.983–1.020)	0.898
METS‐IR Q1	1.00	—	1.00	—	1.00	
METS‐IR Q2	0.961 (0.551–1.674)	0.887	0.749 (0.421–1.334)	0.326	0.972 (0.429–2.201)	0.946
METS‐IR Q3	1.204 (0.705–2.057)	0.496	0.916 (0.523–1.607)	0.760	1.281 (0.560–2.934)	0.557
METS‐IR Q4	2.503 (1.526–4.104)	< 0.001	1.296 (0.739–2.271)	0.365	1.417 (0.605–3.318)	0.421
METS‐IR group trend	1.042 (1.022–1.062)	< 0.001	1.016 (0.994–1.038)	0.163	1.016 (0.984–1.049)	0.334

*Note:* Model 1: unadjusted; Model 2: adjusted for age, sex, disease duration, HBP, and cardiovascular disease; Model 3: adjusted for age, sex, disease duration, HBP, cardiovascular disease, ALT, AST, and eGFR.

TyG‐BMI showed a significant association with HUA in the unadjusted and partially adjusted models when analyzed as a continuous variable; however, this association was attenuated and no longer statistically significant after additional adjustment in Model 3 (OR = 1.002, 95% CI: 0.998–1.006, *p* = 0.289). In quartile analyses, individuals in the highest TyG‐BMI quartile (Q4) had higher odds of HUA compared with Q1 in Model 3 (OR = 2.399, 95% CI: 1.011–5.697, *p* = 0.047), and a significant trend across quartiles was observed (*p* for trend = 0.006). TG/HDL‐C was positively associated with HUA across the regression models. When analyzed as a continuous variable, TG/HDL‐C remained associated with HUA after additional adjustment in Model 3 (OR = 1.120, 95% CI: 1.023–1.225, *p* = 0.013). In quartile analyses, individuals in the highest TG/HDL‐C quartile (Q4) had higher odds of HUA compared with Q1 in Model 3 (OR = 3.309, 95% CI: 1.427–7.671, *p* = 0.005), with a significant linear trend across quartiles (*p* for trend = 0.001). METS‐IR was significantly associated with HUA in the unadjusted model; however, the association was attenuated and became nonsignificant after multivariable adjustment in Models 2 and 3 in both continuous and quartile‐based analyses. To address potential confounding related to the urate‐lowering effects of SGLT2 inhibitors, sensitivity analyses were conducted after excluding participants using SGLT2 inhibitors. The overall patterns and directions of associations between IR surrogate indices and HUA remained similar to the primary analyses, supporting the robustness of the findings (Supporting Information 3: Table S3).

### 3.3. Associations Between IR Surrogates and HUA Stratified by Age

Table 5 presents the associations between IR surrogate indices and HUA stratified by age group (18–44 and 45–59 years). Logistic regression analyses were performed using unadjusted and multivariable‐adjusted models. Among younger participants aged 18–44 years, TyG was associated with HUA in the unadjusted model (Model 1: OR = 1.635, 95% CI: 1.077–2.480, *p* = 0.021); however, this association was attenuated and no longer statistically significant after adjustment in Models 2 and 3. Similarly, TyG‐BMI showed a significant association with HUA in Model 1 (OR = 1.011, 95% CI: 1.004–1.018, *p* = 0.002), but the association was no longer statistically significant after multivariable adjustment. TG/HDL‐C was not significantly associated with HUA across any of the models in the younger group. METS‐IR was associated with HUA only in the unadjusted model (OR = 1.043, 95% CI: 1.015–1.072, *p* = 0.002), but this association was attenuated after adjustment. Overall, none of the IR surrogate indices showed statistically significant associations with HUA after multivariable adjustment in the younger group.

**Table 5:** Association analysis of the insulin resistance index with hyperuricemia in young and middle‐aged people.

Variables	Model 1		Model 2		Model 3	
	OR (95% CI)	*p*	OR (95% CI)	*p*	OR (95% CI)	*p*
**18–44 years**						
TyG	1.635 (1.077–2.480)	0.021	1.369 (0.872–2.148)	0.172	2.577 (0.726–9.155)	0.143
TyG‐BMI	1.011 (1.004–1.018)	0.002	1.006 (0.998–1.014)	0.157	1.006 (0.987–1.025)	0.534
TG/HDL‐C	1.065 (0.991–1.145)	0.088	1.048 (0.976–1.126)	0.198	1.137 (0.819–1.579)	0.443
METS‐IR	1.043 (1.015–1.072)	0.002	1.020 (0.990–1.052)	0.196	1.006 (0.936–1.081)	0.877
**45–59 years**						
TyG	1.625 (1.282–2.060)	< 0.001	1.651 (1.274–2.140)	< 0.001	2.040 (1.332–3.124)	0.001
TyG‐BMI	1.004 (1.000–1.008)	0.040	1.003 (0.999–1.007)	0.098	1.002 (0.997–1.006)	0.449
TG/HDL‐C	1.166 (1.085–1.252)	< 0.001	1.160 (1.076–1.251)	< 0.001	1.187 (1.061–1.327)	0.002
METS‐IR	1.010 (0.996–1.025)	0.161	1.003 (0.987–1.020)	0.684	1.000 (0.981–1.020)	0.975

*Note:* Model 1: unadjusted; Model 2: adjusted for age, sex, disease duration, HBP, and cardiovascular disease; Model 3: adjusted for age, sex, disease duration, HBP, cardiovascular disease, ALT, AST, and eGFR.

In the middle‐aged group (45–59 years), TyG showed a positive association with HUA across the regression models. Specifically, TyG was associated with HUA in Model 1 (OR = 1.625, 95% CI: 1.282–2.060, *p* < 0.001) and Model 2 (OR = 1.651, 95% CI: 1.274–2.140, *p* < 0.001) and remained associated with HUA after additional adjustment in Model 3 (OR = 2.040, 95% CI: 1.332–3.124, *p* = 0.001). TyG‐BMI showed a weak association with HUA in the unadjusted model (OR = 1.004, 95% CI: 1.000–1.008, *p* = 0.040), but this association was no longer statistically significant after adjustment. TG/HDL‐C was associated with HUA in the middle‐aged group across the regression models, including Model 3 (OR = 1.187, 95% CI: 1.061–1.327, *p* = 0.002). METS‐IR was not significantly associated with HUA in any of the models in this age group.

Overall, age‐stratified analyses suggested that the associations between IR surrogate indices and HUA were generally stronger in middle‐aged participants than in younger individuals. In particular, TyG and TG/HDL‐C remained associated with HUA after multivariable adjustment among participants aged 45–59 years, whereas no IR surrogate index showed a statistically significant association with HUA in the younger group after adjustment. To further evaluate the potential confounding effect of SGLT2 inhibitor use, sensitivity analyses were conducted after excluding participants receiving SGLT2 inhibitors. The direction and magnitude of the observed associations were largely consistent with those of the primary analyses, supporting the robustness of the findings (Supporting Information 4: Table S4).

### 3.4. Discriminatory Ability of IR Surrogates for HUA

Table 6 presents the discriminatory ability of IR surrogates for HUA in patients with T2DM, stratified by age and sex. In patients aged 18–44 years, the optimal cutoff value for TyG was 8.79, with a sensitivity of 0.452 and a specificity of 0.847 (AUC = 0.663, 95% CI: 0.557–0.769, *p* = 0.004). In the 45–59‐year group, the TyG cutoff value was 7.82, yielding a sensitivity of 0.632 and a specificity of 0.563 (AUC = 0.609, *p* < 0.001). TyG‐BMI showed the highest AUC among patients aged 18–44 years (AUC = 0.715, 95% CI: 0.610–0.821, *p* < 0.001), with a sensitivity of 0.718 and a specificity of 0.694 at a cutoff value of 226.91. However, this level of discrimination should be interpreted as limited to moderate and does not support the use of TyG‐BMI as a standalone diagnostic or screening tool. Instead, its performance suggests, at most, a potential role as an adjunctive marker for preliminary risk stratification in younger patients. In contrast, the discriminatory ability of TyG‐BMI was lower in patients aged 45–59 years (AUC = 0.601). For TG/HDL‐C, sensitivity was relatively high in patients aged 18–44 years (0.816), with an AUC of 0.671 (95% CI: 0.575–0.768, *p* = 0.001), whereas in patients aged 45–59 years, TG/HDL‐C demonstrated higher specificity (0.761). METS‐IR demonstrated moderate discrimination in patients aged 18–44 years (AUC = 0.698, 95% CI: 0.590–0.807, *p* = 0.001), with sensitivity and specificity values of 0.615 and 0.764, respectively. Overall, IR surrogates tended to exhibit higher AUC values in male patients than in female patients (TyG: 0.653; TyG‐BMI: 0.661; TG/HDL‐C: 0.642; METS‐IR: 0.607); however, the overall discriminatory ability remained limited across both sexes. Among younger participants (18–44 years), TyG‐BMI showed relatively higher discrimination, whereas TG/HDL‐C demonstrated relatively higher specificity in the middle‐aged group (45–59 years). Sensitivity analyses excluding participants receiving SGLT2 inhibitors yielded broadly similar AUC values, cutoff points, and patterns of sensitivity and specificity, indicating that the observed discriminatory ability of IR surrogates was robust to the exclusion of SGLT2 inhibitor users (Supporting Information 5: Table S5).

**Table 6:** ROC‐derived optimal thresholds of insulin resistance surrogate indices for hyperuricemia in patients with T2DM.

Incidence of total hyperuricemia	Cutoff point	Sensitivity	Specificity	AUC	95% CI	*p*
**TyG**						
18–44 years	8.79	0.452	0.847	0.663	0.557–0.769	0.004
45–59 years	7.82	0.632	0.563	0.609	0.549–0.669	< 0.001
Male	7.82	0.714	0.539	0.653	0.596–0.709	< 0.001
Female	8.2	0.5	0.722	0.585	0.453–0.719	0.151
Total	7.82	0.698	0.537	0.640	0.589–0.692	< 0.001
**TyG-BMI**						
18–44 years	226.91	0.718	0.694	0.715	0.610–0.821	< 0.001
45–59 years	179.35	0.784	0.431	0.601	0.543–0.659	0.001
Male	221.57	0.465	0.803	0.661	0.604–0.718	< 0.001
Female	184.91	0.692	0.476	0.556	0.430–0.681	0.353
Total	184.23	0.768	0.469	0.641	0.589–0.693	< 0.001
**TG/HDL-C**						
18–44 years	2.23	0.816	0.511	0.671	0.575–0.768	0.001
45–59 years	2.43	0.441	0.761	0.615	0.558–0.673	< 0.001
Male	2.37	0.558	0.691	0.642	0.588–0.696	< 0.001
Female	2.64	0.448	0.809	0.600	0.475–0.726	0.077
Total	2.3	0.557	0.704	0.645	0.597–0.694	< 0.001
**METS-IR**						
18–44 years	63.68	0.615	0.764	0.698	0.590–0.807	0.001
45–59 years	47.76	0.629	0.498	0.562	0.504–0.622	0.035
Male	57.55	0.419	0.773	0.607	0.547–0.667	< 0.001
Female	45.07	0.731	0.411	0.554	0.428–0.681	0.363
Total	57.21	0.4	0.785	0.608	0.555–0.661	< 0.001

Sensitivity analyses using sex‐specific definitions of HUA (> 360 *μ*mol/L for women and > 420 *μ*mol/L for men) yielded results that were generally consistent with those of the primary analyses (Supporting Information 6: Table S6). For TyG, the cutoff values were 7.82 in males and 8.09 in females, with comparable AUC values in both sexes (0.653). TyG exhibited higher sensitivity in males (0.714) and higher specificity in females (0.725). For TyG‐BMI, males showed higher specificity (0.803) but lower sensitivity (0.465), whereas females demonstrated higher sensitivity (0.707) and lower specificity (0.603), with similar AUC values (0.661 vs. 0.665). TG/HDL‐C showed comparable cutoff values between sexes, with higher sensitivity observed in males and higher specificity in females. METS‐IR exhibited modest discriminatory ability in both sexes, with slightly higher specificity in males and slightly higher sensitivity in females. These findings suggest sex‐related differences in the balance between sensitivity and specificity, while overall discrimination remained modest.

The Hosmer–Lemeshow test indicated adequate calibration for the logistic regression models based on each IR surrogate index (*p* > 0.05). To further assess model stability, internal validation was performed using bootstrap resampling with 1000 iterations. The bootstrap procedure was applied to logistic regression models containing a single predictor (each IR surrogate index) rather than fully adjusted multivariable models. The reported AUC values reflect the apparent performance of these models; no optimism correction was applied. Model performance was evaluated using predicted probabilities derived from the fitted models, and the results demonstrated stable discrimination and calibration (Supporting Information 7: Table S7). This clarification ensures that the observed AUC values are interpreted appropriately and not overestimated.

## 4. Discussion

In this study, we systematically evaluated the associations between four IR surrogate indices and HUA in young and middle‐aged patients with T2DM. Overall, TyG and TG/HDL‐C showed more stable associations with HUA across the regression models, whereas the association for TyG‐BMI was attenuated after additional adjustment when modeled continuously, although individuals in the highest quartile remained associated with higher odds of HUA. In contrast, the association between METS‐IR and HUA was evident primarily in unadjusted analyses and became nonsignificant after multivariable adjustment. In age‐ and sex‐stratified analyses, heterogeneous patterns were observed. ROC analyses suggested limited discriminatory performance of all indices (AUC < 0.75); TyG‐BMI showed relatively higher AUC values in younger participants, whereas TG/HDL‐C demonstrated relatively higher specificity in middle‐aged participants. AUC values were generally slightly higher in men than in women, although overall discrimination remained modest.

A study conducted in a large, nondiabetic population in the United States reported that elevated TyG, TyG‐BMI, TG/HDL‐C, and METS‐IR were positively associated with HUA. In sex‐stratified analyses, TyG‐BMI and METS‐IR demonstrated relatively higher discriminatory performance in women. Notably, in contrast to these findings, the present study observed that among patients with T2DM, AUC values for IR surrogate indices were generally higher in men than in women. This discrepancy may reflect differences in study populations and underlying metabolic contexts. Compared with nondiabetic populations, men with T2DM tend to exhibit greater visceral adiposity and more pronounced IR, as well as lower plasma lipocalin concentrations, all of which have been linked to adverse cardiometabolic profiles [36]. In addition, previous diabetes‐related studies have reported higher baseline SUA levels and a greater prevalence of metabolic syndrome in men [37], which may amplify the association between IR and HUA in this subgroup. Importantly, interpretation of sex‐specific findings in this study is primarily based on the observed results within our diabetic cohort, while prior studies are considered within their respective population contexts. Together, these findings highlight potential sex‐related heterogeneity in the relationship between IR and HUA across different metabolic states.

Our findings are further supported by previous studies reporting associations between IR surrogate indices and HUA across diverse populations. For instance, Xiong et al. [38] found that the AUC for the TyG index was higher than that of other IR indices in hypertensive patients, with several IR indices associated with HUA. Similarly, Li et al. [39] reported associations between TyG, TyG‐BMI, TG/HDL‐C, and METS‐IR and HUA in patients with hypertension. A cross‐sectional study in northeastern Iran also confirmed associations between these IR indices and HUA [40]. Additionally, Han et al. [41] investigated the relationship between SUA and IR proxies in T2DM patients, finding that TG/HDL‐C performed relatively well. In our study, TG/HDL‐C showed consistent associations with HUA across the regression models, particularly among middle‐aged participants, and also demonstrated relatively higher specificity in ROC analyses for this subgroup. Data from Han et al. [42], based on the China Health and Retirement Longitudinal Study (CHARLS), showed that elevated IR surrogate indices were associated with HUA among participants aged ≥ 45 years. By including both young and middle‐aged patients with T2DM, our study extends these observations and suggests age‐related heterogeneity, with more stable associations after multivariable adjustment observed in the middle‐aged group (45–59 years) than in the younger group (18–44 years).

Traditional biomarkers of lipoprotein metabolism, such as HDL‐C and TG, have long been associated with IR and T2DM events [43]. Prior studies have shown that TG/HDL‐C is a useful noninsulin‐based marker reflecting IR [44]. Evidence also suggests that TG/HDL‐C is associated with impaired *β*‐cell function [45]. Prolonged exposure to high levels of fatty acids, a typical manifestation of lipotoxicity, leads to decreased insulin secretion from *β*‐cells, impaired insulin gene expression, and *β*‐cell apoptosis [46]. In states of IR, TG metabolism and HDL formation are altered, resulting in increased TG and decreased HDL‐C levels [47]. The combination of elevated TG and reduced HDL‐C, reflected by a high TG/HDL‐C ratio, represents a dyslipidemic pattern linked to cardiometabolic risk through mechanisms involving lipotoxicity, inflammation, and endoplasmic reticulum stress [48].

Epidemiological studies have shown that patients with HUA frequently exhibit dyslipidemia, characterized by low HDL‐C levels and elevated TG concentrations [49]. The association between elevated TG levels and HUA may be related to altered free fatty acid metabolism [50]. In addition, evidence suggests that HDL‐C plays an important role in glucose metabolism, skeletal muscle glucose uptake, and *β*‐cell insulin secretion [51]. As a protective factor against atherosclerosis, HDL‐C contributes to the attenuation of chronic systemic inflammation and endothelial dysfunction, processes linked to vascular injury and elevated SUA levels [52]. Moreover, elevated SUA concentrations have been associated with diabetes [53]. The findings of the present study are consistent with these observations.

TyG is widely used as a surrogate marker of IR, and recent studies have suggested that TyG‐BMI may better capture IR by incorporating adiposity information [54]. In the present study, TyG showed positive associations with HUA across the regression models, whereas TyG‐BMI demonstrated attenuation after additional adjustment when modeled continuously, despite a persistent association in the highest quartile. Notably, in Model 3, after further adjustment for hepatic and renal function markers (ALT, AST, and eGFR), the OR for TyG increased substantially. This pattern may reflect a suppression effect, in which some metabolically related covariates obscure the true association between TyG and HUA when unadjusted, or it may arise from complex interrelationships among TyG, hepatic and renal markers, and HUA. Nonlinear or threshold effects of TyG and other metabolic factors may also contribute. Therefore, Model 2 was regarded as the primary analytical model because it accounts for major demographic and clinical confounders while minimizing the risk of overadjustment. In contrast, Model 3, which additionally includes hepatic and renal function markers (ALT, AST, and eGFR), should be considered an exploratory metabolic adjustment model. Because these variables may lie on the causal pathway between IR and HUA or reflect downstream metabolic consequences, the findings from Model 3 should be interpreted cautiously, and the potential for overadjustment or collider bias cannot be excluded. TyG was also positively correlated with SUA, which may be partly explained by increased free fatty acid availability associated with elevated TG levels. These FFAs may promote the reabsorption of fatty acid–bound albumin by proximal tubular epithelial cells, leading to cellular apoptosis, renal injury, reduced uric acid excretion, and subsequent elevation of SUA levels [55]. HUA has also been linked to abdominal obesity, as visceral adipose tissue is rich in xanthine oxidoreductase involved in purine metabolism and uric acid production [56, 57]. Sensitivity analyses excluding participants receiving SGLT2 inhibitors yielded patterns broadly consistent with the primary analyses, suggesting that the main findings were not materially driven by SGLT2 inhibitor use.

A key strength of this study lies in its systematic evaluation of IR surrogate indices in relation to HUA while examining heterogeneity by age and sex. However, several limitations should be acknowledged. First, due to the cross‐sectional design, causal or temporal relationships between IR surrogate indices and HUA could not be established. Second, SUA levels were assessed using a single baseline measurement obtained from routine clinical testing, which may introduce some degree of misclassification. Third, the number of HUA events in the younger subgroup (18–44 years) was relatively limited compared with the number of covariates included in the multivariable models. Consequently, model instability, potential overfitting, and reduced statistical power cannot be excluded. Therefore, the findings in this subgroup should be regarded as exploratory and interpreted cautiously. Moreover, the absence of statistically significant associations after multivariable adjustment may partly reflect limited statistical power rather than a true lack of association. Future studies with larger sample sizes are needed to validate these age‐specific findings. Fourth, although multiple covariates were considered, lifestyle‐related factors such as diet, physical activity, smoking, alcohol consumption, and other environmental influences were not comprehensively assessed. In addition, although sensitivity analyses excluding participants receiving SGLT2 inhibitors were conducted, residual confounding related to medication use cannot be entirely ruled out, which may influence the observed associations and their interpretation. Finally, the study included only patients with T2DM, which may limit generalizability to other metabolic conditions. Future multicenter longitudinal studies with more diverse populations are warranted to clarify temporal relationships and to further evaluate the clinical utility of these indices as adjunctive markers for metabolic risk stratification.

## 5. Conclusions

In conclusion, in this cross‐sectional study of young and middle‐aged patients with T2DM, TyG and TG/HDL‐C showed relatively stable associations with HUA across the regression models. Among middle‐aged patients, both the TyG index and the TG/HDL‐C ratio remained associated with higher odds of HUA after multivariable adjustment, with TG/HDL‐C exhibiting relatively higher specificity. Overall, these findings suggest that these indices are not suitable as standalone screening or diagnostic tools but may serve as adjunctive markers for preliminary risk stratification. Prospective longitudinal studies are needed to further clarify the temporal relationship between IR and HUA.

**Nomenclature**
HUA: hyperuricemia
NHUA: nonhyperuricemia
T2DM: Type 2 diabetes mellitus
IR: insulin resistance
TyG: triglyceride–glucose index
URAT1: uric acid transporter Protein 1
GLUT9: glucose transporter Protein 9
SUA: serum uric acid
HOMA‐IR: homeostasis model assessment of insulin resistance
BMI: body mass index
METS‐IR: metabolic score for insulin resistance
BP: blood pressure
HBP: history of hypertension
SBP: systolic blood pressure
DBP: diastolic blood pressure
FBG: fasting blood glucose
2h‐BG: 2‐h postprandial blood glucose
TC: total cholesterol
LDL‐C: low‐density lipoprotein cholesterol
HDL‐C: high‐density lipoprotein cholesterol
TG: triglyceride
HbA1c: glycated hemoglobin
F‐ins: fasting insulin
F‐CP: fasting C‐peptide
BUN: blood urea nitrogen
eGFR: estimated glomerular filtration rate
CVD: cardiovascular disease
FFA: free fatty acid

## Author Contributions

All authors contributed substantially to this work. Hongping Wang drafted the manuscript. Yunyi Yang and Jie Gao prepared tables and figures. Juan Xia, Cuiping Zhang, and Tian Shen collected and rechecked the data. Tao Lei contributed to the discussion; Jun Lu and Zongjun Liu designed and revised the article. Hongping Wang, Yunyi Yang, and Jie Gao contributed equally to this work.

## Funding

This study was supported by the Research Project of Shanghai Municipal Health Care Commission (202240309), the Science and Technology Innovation Project of Shanghai Putuo District Health System (ptkwws202217, ptkwws202302, and ptkwws202417), the Clinical Characteristic of Health System in Putuo District, Shanghai (2024tszk02), the Health System Independent Innovation Science Foundation of Shanghai Putuo District (2023ysxk01), and the Putuo Inheritance Studio of Renowned TCM Physician Chen Yuelai, Shanghai (ptzygzs2409).

## Ethics Statement

The study protocol adhered to the ethical principles outlined in the Declaration of Helsinki and was approved by the Research Ethics Committee of Putuo Hospital, Shanghai University of Traditional Chinese Medicine (Ethics Code: PTEC‐A‐2024‐73(S)‐1).

## Conflicts of Interest

The authors declare no conflicts of interest.

## Supporting Information

Additional supporting information can be found online in the Supporting Information section.

## Supporting information


**Supporting Information 1.** Table S1: Worked example of IR index calculation.

**Supporting Information 2.** Table S2: Association between quartiles of insulin resistance surrogate indices and hyperuricemia (excluding SGLT2 inhibitor users).

**Supporting Information 3.** Table S3: Association analysis of the insulin resistance index with hyperuricemia in young and middle‐aged people (excluding SGLT2 inhibitor users).

**Supporting Information 4.** Table S4: ROC‐derived optimal thresholds of insulin resistance surrogate indices for hyperuricemia in patients with T2DM (excluding SGLT2 inhibitor users).

**Supporting Information 5.** Table S5: Sex‐specific sensitivity and specificity of insulin resistance surrogates for hyperuricemia.

**Supporting Information 6.** Table S6: Calibration and bootstrap internal validation of logistic regression models based on individual insulin resistance surrogate indices for predicting hyperuricemia.

**Supporting Information 7.** Table S7: Calibration and bootstrap internal validation of logistic regression models based on individual insulin resistance surrogate indices for predicting hyperuricemia.

**Supporting Information 8.** Figure S1: Flow diagram of the research process.

## Data Availability

Data will be available to any researcher who contacts the corresponding authors.
