# Supplementary material for: Associations Between a Surrogate Index of Insulin Resistance and Hyperuricemia in Young and Middle‐Aged Patients With Type 2 Diabetes Mellitus
Source: J Diabetes Res. 2026 Jul 2;2026:6682372. doi: 10.1155/jdr/6682372 (PMC13324239; doi:10.1155/jdr/6682372)
Supplement: Supplementary file 1 — Supporting Information 1. Table S1: Worked example of IR index calculation. [file JDR-2026-6682372-s007.docx]

**Supplementary Table S1.**

Worked Example of IR Index Calculation

Unit conversion: FPG (mg/dL) /18 = mmol/L; TG (mg/dL) /88.57 = mmol/L; HDL-C (mg/dL) /38.67 = mmol/L.

| Example patient values |  |
| --- | --- |
| - FPG = 5.5 mmol/L → 99.0 mg/dL | 5.5 × 18 |
| - TG = 1.7 mmol/L → 150.6 mg/dL | 1.7 × 88.57 |
| - HDL-C = 1.2 mmol/L → 46.4 mg/dL | 1.2 × 38.67 |
| - BMI = 25.0 kg/m² |  |
| Formulas and results: |  |
| - TyG = ln[(TG × FPG / 2)] = ln[(150.6 × 99.0)/2] = ln(7447.7) = 8.91 |  |
| - TyG-BMI = ln[(TG × FPG / 2)] × BMI = 8.91 × 25 = 222.7 |  |
| - TG/HDL-C = TG / HDL-C = 150.6 / 46.4 = 3.25 |  |
| - METS-IR = ln[2 × FPG + TG] × BMI / ln(HDL-C) = ln(2 × 99.0 + 150.6) × 25 / ln(46.4) = ln(348.6) × 25 / 3.84 = 5.85 × 25 / 3.84 = 38.1 |  |
