# Supplementary material for: Associations Between a Surrogate Index of Insulin Resistance and Hyperuricemia in Young and Middle‐Aged Patients With Type 2 Diabetes Mellitus
Source: J Diabetes Res. 2026 Jul 2;2026:6682372. doi: 10.1155/jdr/6682372 (PMC13324239; doi:10.1155/jdr/6682372)
Supplement: Supplementary file 2 — Supporting Information 2. Table S2: Association between quartiles of insulin resistance surrogate indices and hyperuricemia (excluding SGLT2 inhibitor users). [file JDR-2026-6682372-s006.docx]

**Table S2. Variance Inflation Factors (VIF) for Covariates in Model 3 Across Four Insulin Resistance Surrogate Indices**

| **Variable** | **TyG** | **TyG-BMI** | **TG/HDL-C** | **METS-IR** |
| --- | --- | --- | --- | --- |
| Age | 1.431 | 1.415 | 1.432 | 1.450 |
| Sex | 1.073 | 1.071 | 1.077 | 1.079 |
| Diabetes duration | 1.342 | 1.323 | 1.323 | 1.318 |
| HBP | 1.224 | 1.236 | 1.218 | 1.233 |
| CVD | 1.179 | 1.165 | 1.175 | 1.164 |
| eGFR | 1.036 | 1.037 | 1.035 | 1.037 |
| ALT | 2.234 | 2.237 | 2.224 | 2.249 |
| AST | 2.194 | 2.196 | 2.162 | 2.181 |
| IR Index | 1.167 | 1.159 | 1.059 | 1.207 |
