# Supplementary material for: Associations Between a Surrogate Index of Insulin Resistance and Hyperuricemia in Young and Middle‐Aged Patients With Type 2 Diabetes Mellitus
Source: J Diabetes Res. 2026 Jul 2;2026:6682372. doi: 10.1155/jdr/6682372 (PMC13324239; doi:10.1155/jdr/6682372)
Supplement: Supplementary file 3 — Supporting Information 3. Table S3: Association analysis of the insulin resistance index with hyperuricemia in young and middle‐aged people (excluding SGLT2 inhibitor users). [file JDR-2026-6682372-s008.docx]

**Table S3. Association between quartiles of insulin resistance surrogate indices and hyperuricemia (excluding SGLT2 inhibitor users)**

| Variables | Model 1 | | | Model 2 | | | | Model 3 | |  |
| --- | --- | --- | --- | --- | --- | --- | --- | --- | --- | --- |
|  | *OR (95% CI)* | *P* | *OR (95% CI)* | | *P* | | *OR (95% CI)* | | *P* | |
| TyG | 1.735 (1.400-2.150) | <0.001 | 1.519 (1.198-1.927) | | <0.001 | | 1.832 (1.256-2.670) | | 0.001 | |
| TyG Q1 | 1.00 | - | 1.00 | | - | | 1.00 | | - | |
| TyG Q2 | 0.897 (0.470-1.711) | 0.742 | 0.943 (0.486-1.828) | | 0.861 | | 1.166 (0.428-3.178) | | 0.763 | |
| TyG Q3 | 1.802 (1.011-3.214) | 0.045 | 1.738 (0.943-3.202) | | 0.076 | | 2.681 (0.992-7.245) | | 0.051 | |
| TyG Q4 | 3.167 (1.828-5.485) | <0.001 | 2.426 (1.322-4.450) | | 0.004 | | 4.647 (1.721-12.542)) | | 0.002 | |
| TyG group trend | 2.056 (1.536-2.751) | <0.001 | 1.726 (1.254-2.375) | | <0.001 | | 2.503 (1.501-4.175) | | <0.001 | |
| TyG-BMI | 1.007 (1.003-1.011) | <0.001 | 1.003 (1.000-1.007) | | 0.062 | | 1.002 (0.998-1.005) | | 0.429 | |
| TyG-BMI Q1 | 1.00 | - | 1.00 | | - | | 1.00 | | - | |
| TyG-BMI Q2 | 1.060 (0.566-1.984) | 0.856 | 0.955 (0.502-1.816) | | 0.887 | | 0.655 (0.239-1.793) | | 0.410 | |
| TyG-BMI Q3 | 1.451 (0.799-2.633) | 0.221 | 1.227 (0.660-2.281) | | 0.518 | | 1.383 (0.550-3.479) | | 0.490 | |
| TyG-BMI Q4 | 3.205 (1.849-5.557) | <0.001 | 2.197 (1.185-4.071) | | 0.012 | 2.260 (0.841-6.068) | | | 0.105 | |
| TyG-BMI group trend | 1.013 (1.008-1.019) | <0.001 | 1.009 (1.003-1.015) | | 0.004 | 1.011 (1.001-1.021) | | | 0.027 | |
| TG/HDL-C | 1.126 (1.065-1.190) | <0.001 | 1.086 (1.025-1.150) | | 0.004 | 1.110 (1.012-1.217) | | | 0.027 | |
| TG/HDL-C Q1 | 1.00 | - | 1.00 | | - | 1.00 | | | - | |
| TG/HDL-C Q2 | 1.153 (0.638-2.081) | 0.637 | 0.992 (0.540-1.824) | | 0.980 | 1.479 (0.548-3.992) | | | 0.439 | |
| TG/HDL-C Q3 | 1.517 (0.861-2.672) | 0.149 | 1.164 (0.643-2.106) | | 0.616 | 1.683 (0.631-4.489) | | | 0.298 | |
| TG/HDL-C Q4 | 3.507 (2.079-5.915) | <0.001 | 2.265 (1.280-4.007 | | 0.004 | 3.800 (1.461-9.886) | | | 0.006 | |
| TG/HDL-C group trend | 1.441 (1.267-1.638) | <0.001 | 1.292 (1.121-1.490) | | <0.001 | 1.426 (1.138-1.785) | | | 0.002 | |
| METS-IR | 1.023 (1.008-1.038) | 0.002 | 1.007 (0.992-1.021) | | 0.376 | 1.000 (0.980-1.021) | | | 0.983 | |
| METS-IR Q1 | 1.00 | - | 1.00 | | - | 1.00 | | |  | |
| METS-IR Q2 | 0.917 (0.503-1.671) | 0.776 | 0.719 (0.386-1.339) | | 0.298 | 1.253 (0.499-3.144) | | | 0.630 | |
| METS-IR Q3 | 1.147 (0.644-2.043) | 0.642 | 0.860 (0.469-1.577) | | 0.625 | 1.397 (0.550-3.553) | | | 0.482 | |
| METS-IR Q4 | 2.367 (1.393-4.022) | 0.001 | 1.239 (0.676-2.270) | | 0.488 | 1.425 (0.537-3.777) | | | 0.476 | |
| METS-IR group trend | 1.040 (1.019-1.062) | <0.001 | 1.014 (0.990-1.038) | | 0.250 | 1.012 (0.976-1.049) | | | 0.517 | |

Model 1: unadjusted;

Model 2: adjusted for age, sex, disease duration, HBP, cardiovascular disease;

Model 3: adjusted for age, sex, disease duration, HBP, cardiovascular disease, ALT, AST, eGFR.
