# Supplementary material for: Associations Between a Surrogate Index of Insulin Resistance and Hyperuricemia in Young and Middle‐Aged Patients With Type 2 Diabetes Mellitus
Source: J Diabetes Res. 2026 Jul 2;2026:6682372. doi: 10.1155/jdr/6682372 (PMC13324239; doi:10.1155/jdr/6682372)
Supplement: Supplementary file 4 — Supporting Information 4. Table S4: ROC‐derived optimal thresholds of insulin resistance surrogate indices for hyperuricemia in patients with T2DM (excluding SGLT2 inhibitor users). [file JDR-2026-6682372-s005.docx]

**Table S4.** Association analysis of insulin resistance index with hyperuricemia in young and middle-aged people (excluding SGLT2 inhibitor users)

| Variables | Model 1 | | | Model 2 | | | Model 3 | |  |
| --- | --- | --- | --- | --- | --- | --- | --- | --- | --- |
|  | *OR (95% CI)* | *P* | *OR (95% CI)* | | *P* | *OR (95% CI)* | | *P* | |
| **18–44 years** |  |  |  | |  |  | |  | |
| TyG | 1.770 (1.119-2.799) | 0.014 | 1.539 (0.931-2.544) | | 0.093 | 2.575(0.706-8.155) | | 0.183 | |
| TyG-BMI | 1.011 (1.003-1.019) | 0.007 | 1.005 (0.996-1.014) | | 0.305 | 1.028 (0.981-1.077) | | 0.248 | |
| TG/HDL-C | 1.068 (0.993-1.150) | 0.076 | 1.055 (0.980-1.136) | | 0.156 | 1.306 (0.862-1.979) | | 0.207 | |
| METS-IR | 1.044 (1.012-1.078) | 0.007 | 1.017 (0.981-1.054) | | 0.369 | 1.090 (0.904-1.313) | | 0.367 | |
| **45–59 years** |  |  |  | |  |  | |  | |
| TyG | 1.568 (1.213-2.028) | <0.001 | 1.543 (1.163-2.047) | | 0.002 | 1.816 (1.151-2.864) | | 0.010 | |
| TyG-BMI | 1.004 (1.000-1.008) | 0.067 | 1.002 (0.999-1.006) | | 0.188 | 1.001 (0.996-1.005) | | 0.747 | |
| TG/HDL-C | 1.142 (1.057-1.233) | <0.001 | 1.127 (1.038-1.222) | | 0.004 | 1.148 (1.026-1.285) | | 0.016 | |
| METS-IR | 1.010 (0.995-1.025) | 0.183 | 1.002 (0.984-1.020) | | 0.838 | 0.996 (0.974-1.019) | | 0.756 | |

Model 1: unadjusted;

Model 2: adjusted for age, sex, disease duration, HBP, cardiovascular disease;

Model 3: adjusted for age, sex, disease duration, HBP, cardiovascular disease, ALT, AST, eGFR.
