# Supplementary material for: Associations Between a Surrogate Index of Insulin Resistance and Hyperuricemia in Young and Middle‐Aged Patients With Type 2 Diabetes Mellitus
Source: J Diabetes Res. 2026 Jul 2;2026:6682372. doi: 10.1155/jdr/6682372 (PMC13324239; doi:10.1155/jdr/6682372)
Supplement: Supplementary file 5 — Supporting Information 5. Table S5: Sex‐specific sensitivity and specificity of insulin resistance surrogates for hyperuricemia. [file JDR-2026-6682372-s004.docx]

**Table S5.** ROC-derived optimal thresholds of insulin resistance surrogate indices for hyperuricemia in patients with T2DM (excluding SGLT2 inhibitor users)

| Incidence of total hyperuricemia | *Cutoff point* | *Sensitivity* | *Specificity* | *AUC* | *95% CI* | *P* |
| --- | --- | --- | --- | --- | --- | --- |
| **TyG** |  |  |  |  |  |  |
| 18-44 years | 8.79 | 0.500 | 0.825 | 0.696 | 0.582-0.811 | 0.002 |
| 45-59 years | 8.08 | 0.495 | 0.694 | 0.602 | 0.539-0.666 | 0.001 |
| Male | 7.82 | 0.720 | 0.543 | 0.650 | 0.589-0.711 | <0.001 |
| Female | 8.43 | 0.409 | 0.824 | 0.585 | 0.437-0.733 | 0.189 |
| Total | 8.06 | 0.594 | 0.650 | 0.639 | 0.583-0.695 | <0.001 |
| **TyG-BMI** |  |  |  |  |  |  |
| 18–44 years | 247.20 | 0.689 | 0.761 | 0.723 | 0.602-0.845 | 0.001 |
| 45-59 years | 179.34 | 0.782 | 0.440 | 0.599 | 0.538-0.661 | 0.002 |
| Male | 184.71 | 0.768 | 0.484 | 0.651 | 0.589-0.713 | <0.001 |
| Female | 182.00 | 0.727 | 0.441 | 0.564 | 0.423-0.706 | 0.317 |
| Total | 184.71 | 0.753 | 0.483 | 0.635 | 0.579-0.691 | <0.001 |
| **TG/HDL-C** |  |  |  |  |  |  |
| 18–44 years | 2.22 | 0.842 | 0.467 | 0.678 | 0.571-0.785 | 0.002 |
| 45-59 years | 2.43 | 0.423 | 0.759 | 0.606 | 0.545-0.667 | <0.001 |
| Male | 2.24 | 0.572 | 0.665 | 0.635 | 0.576-0.693 | <0.001 |
| Female | 3.51 | 0.360 | 0.897 | 0.590 | 0.456-0.724 | 0.136 |
| Total | 2.24 | 0.550 | 0.694 | 0.638 | 0.585-0.691 | <0.001 |
| **METS-IR** |  |  |  |  |  |  |
| 18–44 years | 64.37 | 0.689 | 0.761 | 0.712 | 0.588-0.835 | 0.001 |
| 45-59 years | 47.75 | 0.613 | 0.759 | 0.606 | 0.545-0.667 | 0.042 |
| Male | 63.25 | 0.296 | 0.886 | 0.600 | 0.535-0.665 | 0.002 |
| Female | 60.08 | 0.318 | 0.873 | 0.563 | 0.419-0.707 | 0.328 |
| Total | 60.97 | 0.323 | 0.861 | 0.605 | 0.547-0.663 | <0.001 |
