# Supplementary material for: Associations Between a Surrogate Index of Insulin Resistance and Hyperuricemia in Young and Middle‐Aged Patients With Type 2 Diabetes Mellitus
Source: J Diabetes Res. 2026 Jul 2;2026:6682372. doi: 10.1155/jdr/6682372 (PMC13324239; doi:10.1155/jdr/6682372)
Supplement: Supplementary file 6 — Supporting Information 6. Table S6: Calibration and bootstrap internal validation of logistic regression models based on individual insulin resistance surrogate indices for predicting hyperuricemia. [file JDR-2026-6682372-s001.docx]

**Supplementary Table S6.**

Sex-Specific Sensitivity Analysis for Predicting Hyperuricemia Using IR Surrogates

(420 μmol/L for males and 360 μmol/L for females)

| Incidence of total hyperuricemia | *Cutoff point* | *Sensitivity* | *Specificity* | *AUC* | *95% CI* | *P* |
| --- | --- | --- | --- | --- | --- | --- |
| **TyG** |  |  |  |  |  |  |
| Male | 7.82 | 0.714 | 0.539 | 0.653 | 0.596-0.709 | <0.001 |
| Female | 8.09 | 0.583 | 0.725 | 0.653 | 0.570-0.736 | <0.001 |
| **TyG-BMI** |  |  |  |  |  |  |
| Male | 221.57 | 0.465 | 0.803 | 0.661 | 0.604-0.718 | <0.001 |
| Female | 190.54 | 0.707 | 0.603 | 0.665 | 0.584-0.746 | <0.001 |
| **TG/HDL-C** |  |  |  |  |  |  |
| Male | 2.37 | 0.558 | 0.691 | 0.642 | 0.588-0.696 | <0.001 |
| Female | 2.41 | 0.50 | 0.806 | 0.637 | 0.554-0.720 | 0.001 |
| **METS-IR** |  |  |  |  |  |  |
| Male | 57.55 | 0.419 | 0.773 | 0.607 | 0.547-0.667 | <0.001 |
| Female | 52.92 | 0.431 | 0.774 | 0.609 | 0.523-0.695 | 0.011 |
