# Supplementary material for: Associations Between a Surrogate Index of Insulin Resistance and Hyperuricemia in Young and Middle‐Aged Patients With Type 2 Diabetes Mellitus
Source: J Diabetes Res. 2026 Jul 2;2026:6682372. doi: 10.1155/jdr/6682372 (PMC13324239; doi:10.1155/jdr/6682372)
Supplement: Supplementary file 7 — Supporting Information 7. Table S7: Calibration and bootstrap internal validation of logistic regression models based on individual insulin resistance surrogate indices for predicting hyperuricemia. [file JDR-2026-6682372-s002.docx]

**Supplementary Table S7.**

Calibration and Bootstrap Internal Validation of Logistic Regression Models Based on Individual Insulin Resistance Surrogate Indices for Predicting Hyperuricemia

|  | Hosmer–Lemeshow test | | Bootstrap validation | | | | |
| --- | --- | --- | --- | --- | --- | --- | --- |
|  | Chi-square | *P* | *Sensitivity* | *Specificity* | AUC | 95％ CI | *P* |
| TyG | 9.103 | 0.334 | 0.744 | 0.806 | 0.829 | 0.780-0.878 | <0.001 |
| TyG-BMI | 12.329 | 0.137 | 0.795 | 0.723 | 0.802 | 0.747-0.856 | <0.001 |
| TG/HDL-C | 15.352 | 0.053 | 0.783 | 0.761 | 0.818 | 0.768-0.867 | <0.001 |
| METS-IR | 11.448 | 0.178 | 0.784 | 0.743 | 0.799 | 0.744-0.854 | <0.001 |
