# Supplementary figures and images for: Associations Between a Surrogate Index of Insulin Resistance and Hyperuricemia in Young and Middle‐Aged Patients With Type 2 Diabetes Mellitus
Source: J Diabetes Res. 2026 Jul 2;2026:6682372. doi: 10.1155/jdr/6682372 (PMC13324239; doi:10.1155/jdr/6682372)

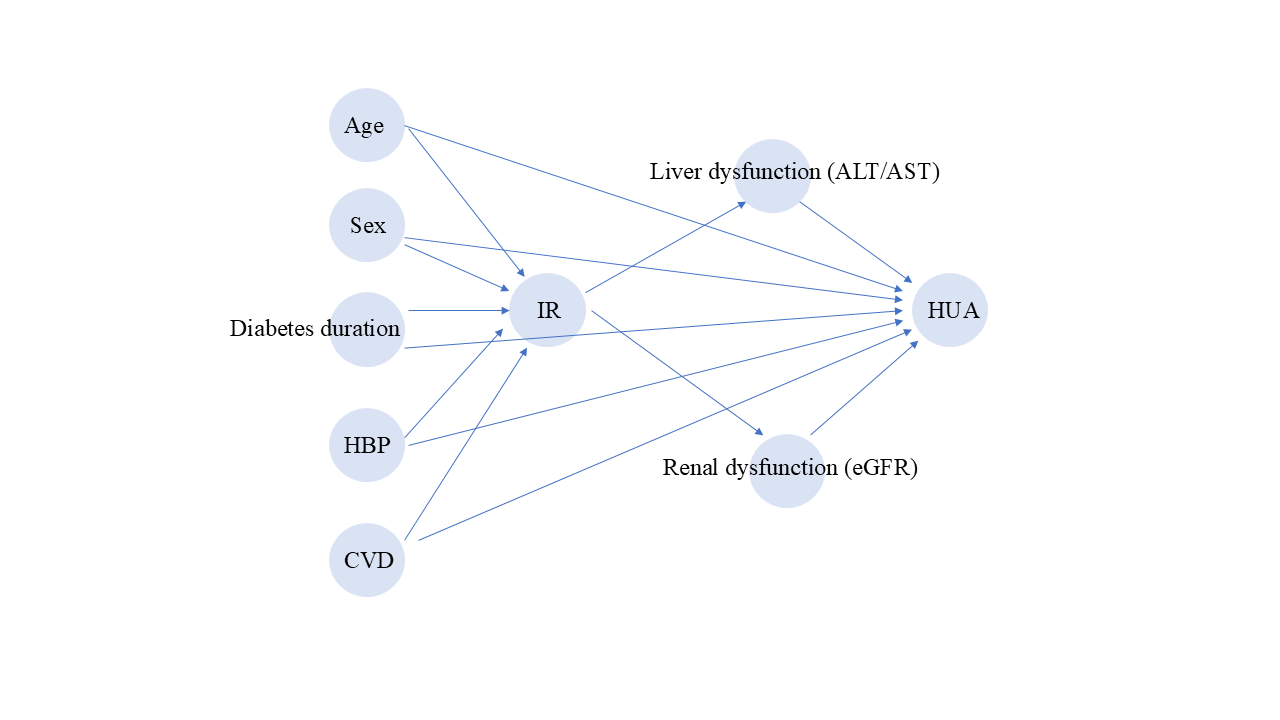

Supplement: Supplementary file 8 — Supporting Information 8. Figure S1: Flow diagram of the research process. [file JDR-2026-6682372-s003.png]
